# What is the effectiveness of community-based health promotion campaigns on chlamydia screening uptake in young people and what barriers and facilitators have been identified? A mixed-methods systematic review

**DOI:** 10.1136/sextrans-2021-055142

**Published:** 2021-08-26

**Authors:** Emma Pearce, Kate Jolly, Isobel Marion Harris, Ada Adriano, David Moore, Malcolm Price, Jonathan Ross

**Affiliations:** 1 Institute of Applied Health Research, University of Birmingham, Birmingham, UK; 2 Institute of Microbiology and Infection, University of Birmingham, Birmingham, UK

**Keywords:** chlamydia infections, health promotion, public health

## Abstract

**Background:**

The UK National Chlamydia Screening Programme uses an opportunistic approach. Many programmes use campaigns to raise awareness of chlamydia screening in young people. This review aimed to assess the effectiveness of campaigns on uptake of chlamydia screening in young people.

**Methods:**

We conducted a mixed-methods systematic review of articles assessing the outcomes of community-based health-promotion campaigns to increase chlamydia screening in young people, their experiences of the campaigns and other facilitators and barriers to the conduct of the campaigns. We searched four databases for quantitative and qualitative studies with no language restrictions.

**Main results:**

From 10 329 records identified, 19 studies (20 articles) were included in the review: 14 quantitative, 2 qualitative and 3 mixed methods. All studies with quantitative outcomes were before-after study designs or interrupted time series. The prediction interval for relative change (RC) in test counts ranged from 0.95 to 1.56, with a summary pooled estimate of RC 1.22 (95% CI 1.14 to 1.30, 13 studies, I^2^=97%). For test positivity rate, 95% prediction interval was 0.59 to 1.48, with a summary pooled estimate of RC 0.93 (95% CI 0.81 to 1.07, 8 studies, I^2^=91.8%). Large variation in characteristics between studies precluded exploring outcomes by type of campaign components. Seven major qualitative themes to improve screening were identified: targeting of campaigns; quality of materials and message; language; anonymity; use of technology; relevance; and variety of testing options.

**Conclusions:**

Health promotion campaigns aiming to increase chlamydia testing in those aged 15–24 years may show some effectiveness in increasing overall numbers of tests, however numbers of positive tests do not follow the same trend. Qualitative findings indicate that campaigns require clear, relevant messaging that displays the full range of testing options and assures anonymity in order to be effective.

## Introduction

*Chlamydia trachomatis* (CT) is the most common bacterial STI in the UK and worldwide, with reported prevalence up to 12%.^1^ It disproportionately affects adolescents, some ethnic minorities and those with lower socioeconomic status.^2 3^ In women, CT infection increases the risk of pelvic inflammatory disease, ectopic pregnancy and infertility.^4^

Many countries have implemented screening programmes focused on those aged under 25 years.^5 6^ Register-based screening for CT is likely to be of limited effectiveness, unlike with screening for many cancers,^7^ so opportunistic CT screening using health promotion methods tends to be more favoured. This opportunistic approach can present drawbacks, including increased screening of the ‘worried well’.^8^

Methods used to promote CT screening vary widely, and no one method has been shown to be superior to, or more cost-effective than, others.^9^ There may be value in face-to-face health promotion campaigning, and also in underpinning screening campaigns with social marketing techniques.^10^ How to reach young people effectively remains uncertain; the move towards using social media to support health promotion campaigns may increase reach and engagement, but risks lack of information clarity, or inability to accurately monitor how the information is used.^11^

### Study aim

This review aimed to answer two questions:

1. Which community-based health promotion campaign methodologies are most effective in promoting chlamydia screening to those aged 15–24 years?
2. What facilitators and barriers of these campaign methods exist for the campaign teams providing the service and the young people responding to the campaigns?

## Methods

This review was prospectively registered on PROSPERO CRD42020169288.^12^

### Data sources and searches

Joanna Briggs Institute Guidelines on conducting mixed-methods systematic reviews, Preferred Reporting Items for Systematic Review and Meta-Analyses and Enhancing Transparency in Reporting the synthesis of Qualitative research reporting guidelines were followed.^13–15^ Electronic databases searched were MEDLINE, MEDLINE in Process, EMBASE and CINAHL. Open Grey was searched, and requests were made to regional and national public health and sexual health networks across the UK to uncover unpublished evaluations. Reference lists of relevant systematic reviews and included studies were reviewed for further relevant studies.^10 11 16^

Databases were searched using free text and index terms (as appropriate) relating to CT, campaign methods and health promotion terminology (online supplemental appendix 1). Searches were run from inception of each database to week 4 of March 2020, with no restrictions by date, country of origin or publication language. Abstracts and full texts were assessed in duplicate for inclusion by review team members using Covidence software.^17^ All discrepancies were discussed, with additional team members supporting consensus where required.

10.1136/sextrans-2021-055142.supp1 Supplementary data

### Study selection

Inclusion and exclusion criteria for quantitative and qualitative study designs can be seen in table 1.

**Table 1:** Inclusion/Exclusion criteria

Category	Inclusion criteria	Exclusion criteria
Population	All aged 15–24 years; mean/median age between 15 and 24 years; 75% aged under 30 years (category dependent on reporting style within studies)	Workers in the sex industry
		Pregnant participants
Context	Community-based at any level (national, regional, local)	Controlled settings (settings in which the participants have no recourse to leave of their own free will)
		Health promotion campaigns in a single GP practice
Intervention/Exposure	Wholly or mainly community-based health promotion interventions	Health promotion opportunities forming part of a healthcare consultation
Comparator/Control	Quantitative study designs: similar population with no intervention exposure or a formalised control group Qualitative study designs: no control/comparator groups were required	
Study design	Quantitative study designs: randomised controlled trials, non-randomised controlled trials, trials with a concurrent/historical comparator, before-and-after studies, interrupted time series Qualitative study designs: any	All other study designs

This table is not reproduced from another source and has been created by the review team for the current publication.

### Data extraction and quality assessment

Data were extracted onto predesigned, piloted extraction forms. All studies were extracted once, with 10% of studies extracted by a senior reviewer as a consistency check.

Quality assessment for quantitative studies was achieved by adapting the pre-post test study quality assessment tool of the US National Heart, Lung and Blood Institute.^18^ Further information on assessment methodology can be found in the online supplemental file. Quality assessment for qualitative studies was undertaken using the Critical Appraisal Skills Programme qualitative checklist.^19^

10.1136/sextrans-2021-055142.supp4 Supplementary data

Quality assessment of included studies was completed by two reviewers independently.

### Data synthesis and analysis

Narrative synthesis of data from quantitative studies on number of tests, number of positive tests and per cent positivity was undertaken. Only number of tests and per cent positivity are reported here owing to word count limitations; number of positive tests can be found in the online supplemental file. Data on relative change (RC) in test numbers and per cent positivity were meta-analysed using a random effects model. The high heterogeneity in the type of campaign, its specific attributes and the target population mean that the pooled effect should be thought of as an indicative average which cannot be applied directly to any situation. Prediction intervals were calculated to describe the range in which 95% of the distribution of the effects lie. The summary estimates must be considered in light of these prediction intervals, which should be considered the primary results from the synthesis. Quantitative data analysis was completed using STATA V.17.^20^

For qualitative and mixed-methods studies, thematic analysis of extracted data was undertaken by one reviewer and reviewed with another.

Following these separate analyses, a segregated convergent approach was taken when combining quantitative and qualitative information. This created a richer narrative describing effectiveness, acceptability and feasibility.

## Results

### Study selection

**Figure 1:**
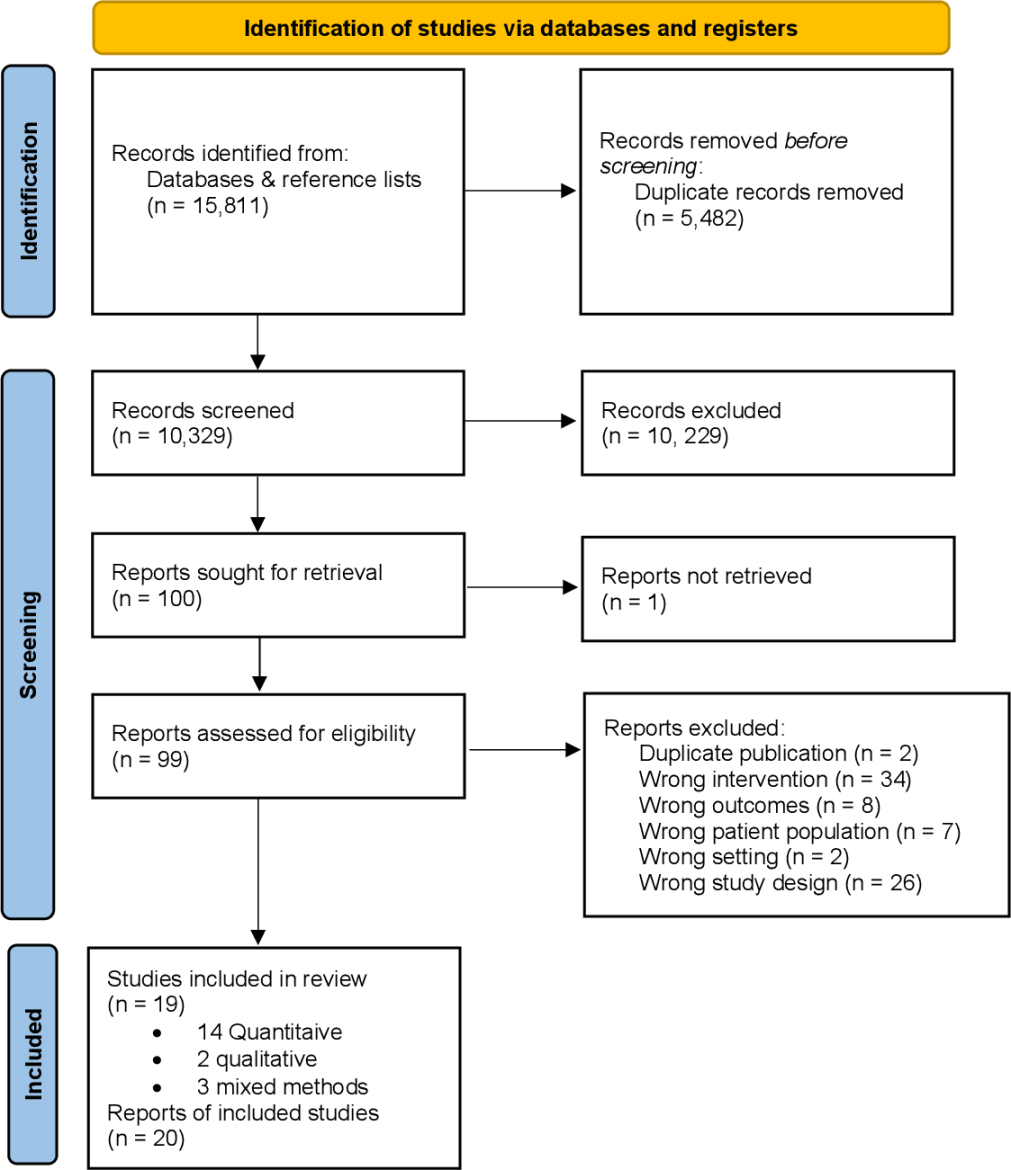
Preferred Reporting Items for Systematic Review and Meta-Analyses flow diagram detailing the search process for included studies. This image is not reproduced from another source and has been created by the review team for the current publication.

Of 10 329 records identified and screened for eligibility, 100 full-text articles were assessed and 19 studies (reported in 20 articles) included (figure 1). Fourteen were quantitative studies,^21–34^ two qualitative^35 36^ and three had mixed-methods elements.^37–39^ All studies were published in English and based in high-income countries: eight conducted in the USA,^21 25 26 32 33 36 38 40 41^ seven in Australia,^22–24 28 30 31 39^ three in England^27 35 37^ and one in Canada.^34^ One study contributed two datasets to the quantitative analysis as it investigated two separate campaigns.^30^

### Quantitative findings

This section discusses the 17 studies with quantitative components: 14 solely quantitative and 3 mixed methods.

#### Study and campaign characteristics

Communities/Settings included educational establishments,^21 26^ high-risk populations (men who have sex with men or minority ethnic groups)^22 33 38^ and specific communities or age groups within entire counties or countries. Two campaigns were venue-based,^21 38^ meaning both the campaigning and any subsequent testing occurred at a single location. Six campaigns were partially venue-based,^22 24 26 30 31 41^ with the campaign or the testing strategy (but not both) occurring in a single location. The remaining nine campaigns were truly community-based,^23 25 27 28 32–34 37 39^ with neither the campaign nor the wider testing strategy tied to a specific location.

All campaigns used a mixture of methods including print media, peer supporters and social media (table 2). Some campaigns also referenced specific adaptions made to their materials, such as creating multilingual materials^33 34^ or focusing on high-risk communities.^38^ Four studies made use of the ‘Get Yourself Tested’ campaign,^25 26 32 38^ a US-based public partnership between MTV and the Kaiser Family Foundation. Each of those campaigns targeted different sections of society, including female-only, high-risk youth, one state-wide and one city-wide campaign and different campaign methodologies were chosen from a material ‘bank’ and targeted to effectively reach the population in question. This variation in study characteristics precluded exploring outcomes by type of campaign component.

**Table 2:** Characteristics of included quantitative studies/components

Study	Country	No. participants tested during campaign (see descriptors)	Median age (range)	Campaign length (weeks)	Health promotion campaign methods					
					Print*	Verbal†	Web-based‡	Social media§	Peer¶	Direct**
Anderson *et al* 21	USA	333††	NR (NR)	6	Y	Y	Y	Y	Y	Y
Buhrer-Skinner *et al* 22	Australia	341‡‡	22.6 (19.6–28)	48	Y	N	Y	N	Y	N
Chen *et al* 23	Australia	NR	NR (16–30)	60	Y	N	Y	N	N	N
Debattista *et al* 24	Australia	109‡‡	NR (NR)	39	Y	N	Y	N	N	N
Dowshen *et al* 41	USA	4628‡‡	17.2 (SD±1.86)§§	52	Y	Y	Y	Y	N	N
Friedman *et al* 26	USA	NR	NR (NR)	34	Y	Y	Y	Y	N	Y
Friedman *et al* 25	USA	NR	NR (NR)	104	Y	Y	N	N	Y	Y
Garbers *et al* 38	USA	266††	NR (15–25)	12	Y	N	Y	Y	Y	N
Gobin *et al* 27	England	NR	NR (15–24)	4	Y	Y	Y	N	N	N
Gold *et al* 28	Australia	NR	NR (NR)	4	Y	Y	Y	N	Y	N
Kwan *et al* 30	Australia	159‡‡	16–24	2 and 12	Y	Y	N	N	N	N
Miller *et al* 31	Australia	5516††	NR (15–35)	8	Y	Y	N	N	N	N
Nadarzynski *et al* 37	England	472‡‡	NR (13–24)	NR	N	N	N	Y	N	N
Roston *et al* 32	USA	NR	21.2 (±2.5)§§	4 and 4	Y	Y	N	N	N	N
Rotblatt *et al* 33	USA	1619‡‡	22.3 (12–26)	52	Y	Y	N	N	Y	N
Wackett *et al* 34	Canada	911‡‡	NR (NR)	16	Y	Y	N	N	Y	N
Wilkins and Mak^39^	Australia	NR	NR (18–29)	12	Y	Y	Y	N	N	Y

This table is not reproduced from another source and has been created by the review team for the current publication.

*Print media: posters, newspaper/magazine articles, leaflets, other materials, for example, credit cards, beer mats.

†Verbal media: radio adverts, TV adverts.

‡Web-based: internet sites used mainly for campaigns materials (not just for ordering kits).

§Social media: Facebook, Twitter, Instagram, etc.

¶Peer: engaging peers to deliver campaigning and materials, for example, at University events, in nightclubs.

**Direct: materials direct to personal technology devices, for example, SMS, Facebook push adverts.

††Number screened at event/clinic.

‡‡Kits returned.

§§Mean and SD reported in place of median (range).

N, no/absent; NR, not reported; Y, yes/present.

Outcomes recorded included number of tests, number of positive tests, per cent population tested and per cent positive tests. Data were split by gender where possible (binary variable only owing to study reporting), however most reported combined figures, with number of tests before and after being the most reported outcome (13/17 studies).

#### Quality assessment

**Figure 2:**
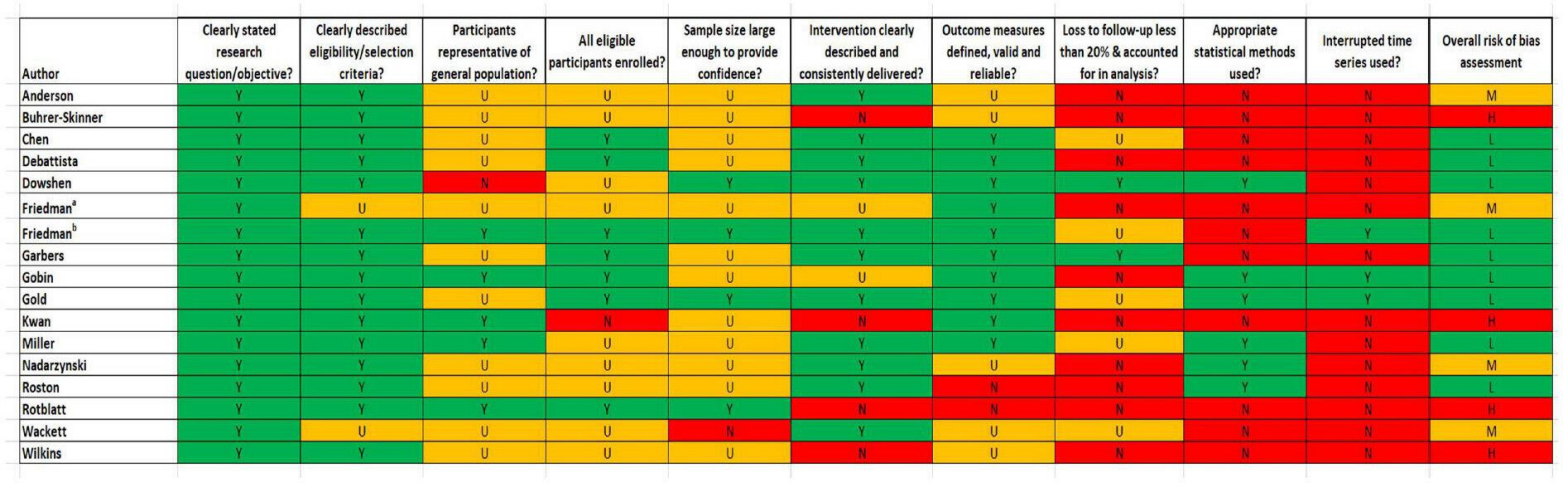
Risk of bias of included quantitative studies. This image is not reproduced from another source and has been created by the review team for the current publication. H, high; L, low; M, medium; N, no; U, unclear; Y, yes.

Nine quantitative studies were assessed as having low,^23–25 27 28 31 32 38 41^ four medium^21 26 34 37^ and four high risk of bias^22 30 33 39^ (figure 2). Following assessment, it was apparent that quantitative studies tended to provide a clear description of the research question, selection criteria and outcome measures, but lack of detail meant that demonstration of enrolling all eligible participants, using appropriate statistical methods or accounting for loss to follow-up were difficult to assess.

#### Study results

##### Number of tests

**Figure 3:**
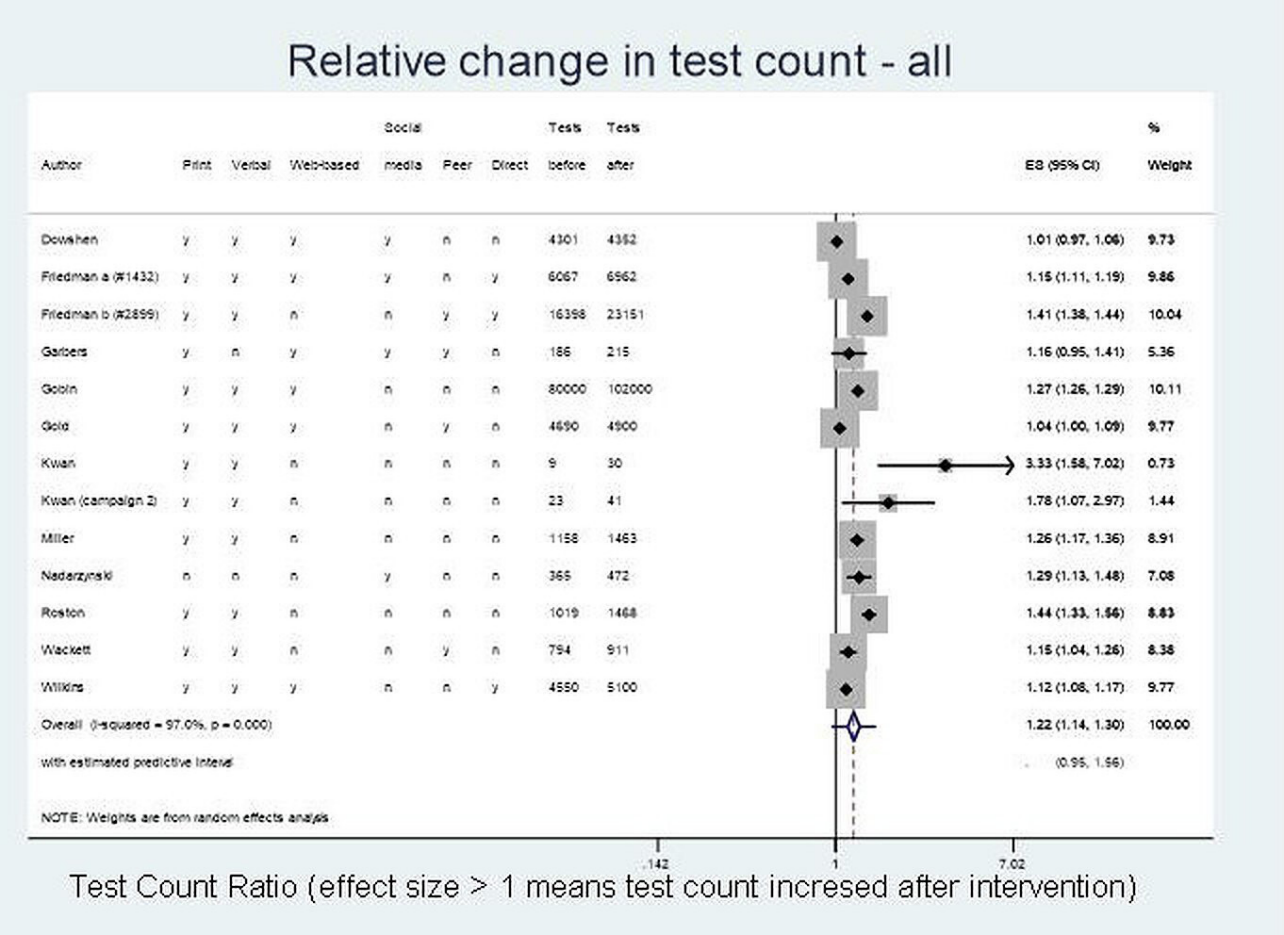
Relative change in test count (all). This image is not reproduced from another source and has been created by the review team for the current publication.

Meta-analysis for RC in test count (13 studies) demonstrated a potential for increase in overall test numbers following a health promotion campaign (RC 1.22; 95% CI 1.14 to 1.30), equating to a potential 22% increase in testing (figure 3). This was countered by extremely high heterogeneity between studies (I^2^=97%, p<0.001). The estimated predictive interval, which is less affected by the extreme heterogeneity, ranges from 0.95 to 1.56, indicating anything from no effect of testing post-campaign to a 56% increase in tests.

There was no evidence of a difference in summary effect between genders; males (four studies) RC=1.26 (95% CI 1.08 to 1.47; I^2^=94%, p<0.001;estimated predictive interval: 0.60 to 2.63) and females (five studies) RC=1.23 (95% CI 1.05 to 1.45; I^2^=95.6%, p<0.001; estimated predictive interval: 0.66 to 2.31) (online supplemental figures 3 and 4). However, the CIs and predictions do not rule out the possibility of important differences overall, or in some settings and/or some campaign types.

#### Positivity rate

**Figure 4:**
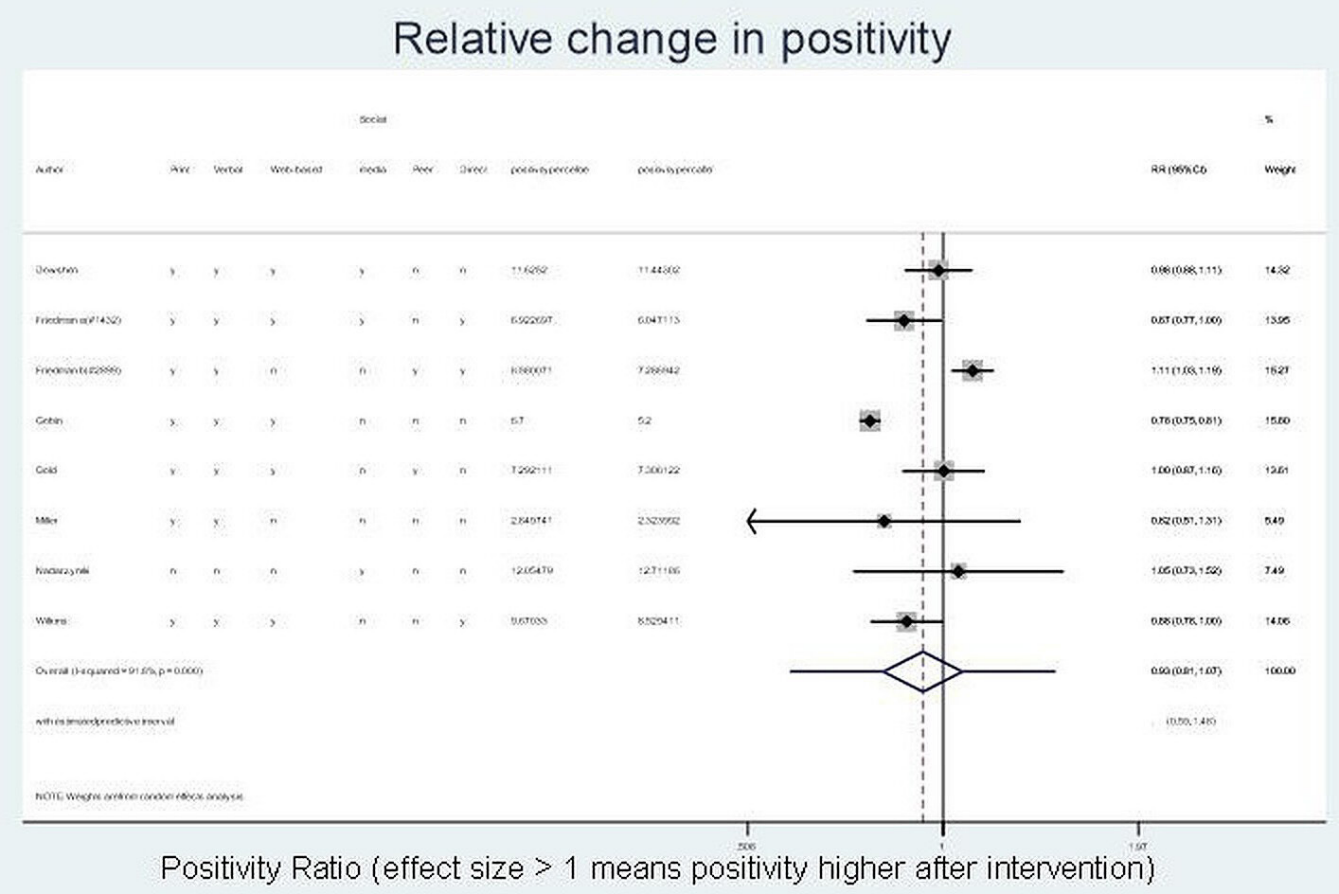
Relative change in per cent positivity (all). This image is not reproduced from another source and has been created by the review team for the current publication.

RC in test positivity was reported or calculable for eight studies. Overall, it was unclear whether there was an effect on test positivity rate following a campaign (RC 0.93; 95% CI 0.81 to 1.07), with high heterogeneity between studies (I^2^=91.8%, p<0.001) (figure 4). The estimated predictive interval demonstrated minimal effect of campaigns on test positivity rate, ranging from 0.59 to 1.48.

For males (four studies), change in positivity rate was similar (RC 0.93 (95% CI 0.68 to 1.26); I^2^=83.3%, p=0.000) with an estimated predictive interval of 0.24 to 3.57; for females (four studies), the estimated change in positivity was slightly higher (RC 1.08; 95% CI 0.90 to 1.31; I^2^=75%, p=0.007) with an estimated predictive interval of 0.50 to 2.35 (online supplemental figures 6 and 7). However, as above, the CIs and predictive intervals do not rule out the possibility of important differences overall, or in some settings and/or some campaign types.

### Qualitative findings

#### Study and campaign characteristics

One qualitative study was undertaken in the USA^36^ and one in England.^35^ The American campaign focused on views of campaign participants, while the English study focused on general practice opportunistic screening provider views. The three mixed-methods evaluations were conducted in Australia,^39^ England^37^ and the USA^38^ and all focused on participant views.

#### Quality assessment of included studies

Of the two included qualitative studies, one was assessed as being at low risk of bias,^35^ and the other, an abstract, at medium risk.^36^ Of the three mixed-methods studies, two were assessed as being at low risk of bias^37 38^ and one at high risk.^39^

#### Qualitative findings

Seven major themes were identified: targeting of campaigns; quality of materials and message; language; anonymity; use of technology; relevance and variety of testing options. Many of these were barrier and/or facilitator to campaign effectiveness, so are addressed as themes in their entirety. All quotations that support themes can be found in online supplemental appendix 3.

10.1136/sextrans-2021-055142.supp3 Supplementary data

##### Targeting

While many campaigns are targeted towards young people to try to increase engagement and testing rates, young people reported that more generic messaging was required. They described the way that targeting could be seen as negative and may disengage young people from testing messages.^38^

##### Quality of message and materials

Young people and staff suggested that high-quality materials were important in increasing testing. High quality was defined by look and clarity of message. Simplistic messages, or gender-specific messages were viewed as patronising by staff and young people.^35 37^

##### Language

Staff said having materials available in a variety of spoken languages would help increase testing rates, and despite cost implications was probably worth pursuing.^35^

##### Anonymity

All groups indicated that anonymity throughout was important—if a young person was seen actively engaging with the campaign materials this may discourage subsequent testing. Campaign materials therefore need to be discreet and easily seen in public places where issues of stigma can be concealed.^37^

##### Use of technology

All groups indicated that the move towards social media usage should be capitalised on by health promotion campaigners in order to bring about increased testing.^35 37^

##### Relevance to the young person

Young people valued relevance of messages, wanting to see themselves reflected in messaging, sometimes through sexual identity and often in wider characteristics; this was linked to comments around making campaign materials more discreet. It was suggested that national campaigns could be linked to popular TV programmes and media.^37 38^

##### Variety of options for testing

A minor theme, noted less frequently than others, was that of ensuring that the full variety of options is publicised effectively. It was felt that campaigns that only directed people towards one testing avenue when multiple exist may highlight problems with access/affordability resulting in lower testing rates.^35^

### Convergence of findings

Targeting was used by all studies included in the quantitative analysis by directing messaging to young people and/or displaying campaign materials in young person-friendly settings. Relevance of message was a technique explicitly used in nine of the quantitative campaigns^25–28 33 34 37 38 41^; this was most commonly achieved by using pictures of representative young people, and reviewing campaign materials with focus groups taken from the target audience prior to the campaign. Anonymity was demonstrated in seven studies, usually by allowing for online requesting with pseudonyms if required.^22 25 26 30 33 37 41^ Those studies that did not clearly offer anonymous testing were generally those that required the participant to attend a medical facility in order to have the CT test, generally forcing reliance on transport from friends/family or unexplained journeys away from home.

Use of personal technology was a less common campaign facilitator (five studies^25 26 37 39 41^), as was clear demonstration of a wide variety of testing options (five studies^22 25 26 38 41^). The use of personal technology has grown in popularity; many included studies were conducted at a time when sole access to a technological device for the young person could not be guaranteed, which could also compromise anonymity. By demonstrating a wide variety of options for testing, campaigns aim to increase the number of tests taken; campaigns generally combine this technique with increased relevance of materials to young people. Just two campaigns produced materials in other languages; one in Canada where both French and English have official federal status^34^ and one in an area of the USA where Spanish is commonly spoken.^33^ Quality of materials could not be assessed within the review as this concept needs to be viewed in the context of the intended audience, and no study commented on quality of materials in this context explicitly.

Online supplemental appendix 2 displays the RC in test outcomes compared with the presence/absence of facilitative factors identified by the qualitative analysis. The convergent findings neither strongly support nor refute the qualitative findings of this review.

10.1136/sextrans-2021-055142.supp2 Supplementary data

## Discussion

### Summary

This review provides some evidence that health promotion campaigns can increase chlamydia testing rates among young people. While there was no evidence that this translates into increased positive tests or positivity rate, this cannot be ruled out entirely. Commonly used campaign methods included posters and radio advertising, as well as social media. Qualitative findings suggest attention should be paid to the quality of campaign materials and the language used, ensuring it's understood by different cultures and does not alienate young people. Engaging with campaigns should also have the option of anonymity, and to this end, increased use of personal technology may be important.

### Strengths and limitations

This review draws on a variety of established methodologies to provide the most comprehensive ascertainment and robust synthesis of quantitative, qualitative and mixed-methods studies available. The questions addressed are important ones, particularly in the context of financial constraints around sexual healthcare in many parts of the world. Owing to the quality of the included papers, as well as time constraints precluding contact with authors, a pragmatic decision was made to include those studies in which mean/median age range could not be calculated; there remains a small risk that these studies contained >10% participants outside of our desired age range.

When discussing quality of included studies, no controlled or randomised studies were found as part of the review process. Population size and characteristics, including gender diversity, were poorly described by many study authors precluding the calculation of directly comparable outcome measures; this should be viewed in the context of challenges defining the population of community-based studies. Post-campaign data are often collected shortly after the end of the campaign meaning long-term effects on testing rates cannot be explored. Many studies were excluded solely on the basis that they lacked pre-test data, reducing the breadth of information that may have informed the review outcome. Outcome measures were heterogeneous, but most studies reported number of tests, enabling some statistical comparison between studies. Campaign methodologies were generally well-described, allowing use of specific techniques to be extracted from the studies. Large variation in characteristics between studies precluded exploring outcomes by type of campaign component. The same issue applies to specific population-targeted campaigns as reporting issues and mixing of methodologies means that no specific conclusions on key components of effectiveness can be identified. There were relatively few studies with qualitative data; one quantitative study had a rich description of the intervention but did not use qualitative methods or report findings in a way that could be extracted as part of this review.^26^

### Interpretation (in light of other evidence)

Population-based screening provides a benefit by reaching populations not ordinarily tested opportunistically or by invitation by clinicians in other settings.^42^ A Netherlands-based study found this method revealed a similar positivity rate to tests conducted within sexual health services and tended to encourage more men and younger age groups to come forward for testing.^42^ Many included campaigns focused on promoting web-based requesting of test kits; this method has been found to be preferred over other ways of accessing testing^43^ and this is supported by our finding that anonymity is important to young people. The demographics of populations accessing CT testing in this way also match those most at risk of infection and complications, namely younger women, and those from minority populations.^44 45^ It must be noted, however, that using technology alone has the potential to widen inequalities of access, particularly among younger men in lower socioeconomic groups, who often access screening at a lower rate.^46^ The messages contained in each campaign also need to be well thought out to have the desired effect on the target population; there is growing evidence that messages highlighting negative differences between two communities may not encourage the desired behaviour.^47^

The effects of health promotion campaigns are often difficult to clearly identify owing to the unintended consequences that this methodology can bring, and difficulties in separating campaign effects from the everyday context of peoples’ lives.^48^ Media-based campaigns for other disease states tend to increase awareness of the need for intervention during the campaign period, but this effect is unlikely to be sustained without continued messaging^49^; this is echoed in some chlamydia screening campaigns, with increased rates of testing at campaign end that quickly return to precampaign levels.^30^ Issues may arise from the types of campaigns used as most public health promotion research tends to focus on passive information-giving centred on individual behaviour change which rely on many elements, including personal health literacy skills, and may ignore more community-centred approaches that support long-term cultural change that is understood by all.^50^ Some of the more successful campaigns in our review attempted to use this approach by taking standard materials and adapting them with community input.^25 26 32 38^ This does not move beyond the potential problem of relying solely on words and pictures to engender behaviour change, however by involving young people in the production of the messages it may start to address differing levels of health literacy and ensure that messages are clearly understood by all which could result in positive behaviour change, such as increased CT testing.^51^

### Practical and research recommendations

Future campaign evaluations should aim to use methods such as cluster RCTs or interrupted time series. Improving description of included populations, campaign elements and breakdown of subgroup elements will aid classification and quality assessment, while focusing on test count and positivity rates will ensure that future studies can be subject to robust meta-analysis. Researchers and policy makers will therefore be supported to make well-informed decisions in an area of healthcare provision which remains contentious. It is important to note that there remains little evidence to support CT screening more generally,^52^ and as such this review aids targeting of screening campaigns, directing scarce resources towards the most effective methods and the populations most likely to benefit.

## Conclusion

Health promotion campaigns aiming to increase chlamydia testing in those aged 15–24 years may show some effectiveness in increasing overall numbers of tests, however there was no evidence that numbers of positive tests follow the same trend, although this cannot be ruled out. Based on current evidence, no specific methodology can be said to be more effective than others, however those that improve anonymity, provide access via multiple languages and display real relevance to the young person by reflecting their reality in the materials used may offer some advantage. Future research needs to focus on improved data collection to address specific questions regarding how to best target at-risk populations and optimise methodologies, while acknowledging uncertainties in more widespread population-based chlamydia screening.

**Key messages**

- Promotional campaigns have some efficacy in increasing the overall number of young people screened for chlamydia.
- Despite this, corresponding increases in test positivity rates are not seen.
- Facilitators for screening include assurances of anonymity, access via multiple languages and perceived identification with campaign materials.

## References

[1] Dielissen PW , Teunissen DAM , Lagro-Janssen ALM . Chlamydia prevalence in the general population: is there a sex difference? A systematic review. BMC Infect Dis 2013;13:534. 10.1186/1471-2334-13-534 24215287 PMC4225722

[2] Public Health England . Sexually transmitted infections and screening for Chlamydia in England, 2017. Available: https://assets.publishing.service.gov.uk/government/uploads/attachment_data/file/713944/hpr2018_AA-STIs_v5.pdf

[3] Crichton J , Hickman M , Campbell R , et al . Socioeconomic factors and other sources of variation in the prevalence of genital Chlamydia infections: a systematic review and meta-analysis. BMC Public Health 2015;15:729. 10.1186/s12889-015-2069-7 26224062 PMC4520210

[4] Price MJ , Ades AE , Soldan K , et al . The natural history of Chlamydia trachomatis infection in women: a multi-parameter evidence synthesis. Health Technol Assess 2016;20:1–250. 10.3310/hta20220 PMC4819202 27007215

[5] Public Health England . NCSP: Programme overview [Internet], 2017. Available: https://www.gov.uk/government/publications/ncsp-programme-overview

[6] Quezada-Yamamoto H , Dubois E , Mastellos N , et al . Primary care integration of sexual and reproductive health services for Chlamydia testing across WHO-Europe: a systematic review. BMJ Open 2019;9:e031644. 10.1136/bmjopen-2019-031644 PMC6803110 31628129

[7] Low N , Redmond S , Uusküla A , et al . Screening for genital chlamydia infection. Cochrane Database Syst Rev 2016;9:CD010866. 10.1002/14651858.CD010866.pub2 27623210 PMC6457643

[8] Buhrer-Skinner M , Muller R , Bialasiewicz S , et al . The check is in the mail: piloting a novel approach to Chlamydia trachomatis testing using self-collected, Mailed specimen. Sex Health 2009;6:163–9. 10.1071/SH08076 19457297

[9] Roberts T , Robinson S , Barton P . Chlamydia screening studies (class) group. screening for Chlamydia trachomatis: a systematic review of the economic evaluations and modelling. Sex Transm Infect 2006;82:193–200. 16731666 10.1136/sti.2005.017517 PMC2593085

[10] Phillipson L , Gordon R , Telenta J , et al . A review of current practices to increase Chlamydia screening in the community--a consumer-centred social marketing perspective. Health Expect 2016;19:5–25. 10.1111/hex.12337 25580560 PMC5055217

[11] Gabarron E , Wynn R . Use of social media for sexual health promotion: a scoping review. Glob Health Action 2016;9:32193. 10.3402/gha.v9.32193 27649758 PMC5030258

[12] Pearce E , Harris B , Adriano A . What is the effectiveness of different community-based health promotion campagin methods on chlamydia screening uptake in young people and what barriers and facilitators have been identified? A mixed-methods systematic review. PROSPERO [Internet], 2020. Available: https://www.crd.york.ac.uk/prospero/display_record.php?ID=CRD42020169288 10.1136/sextrans-2021-055142 PMC8785066 34446545

[13] Tong A , Flemming K , McInnes E , et al . Enhancing transparency in reporting the synthesis of qualitative research: ENTREQ. BMC Med Res Methodol 2012;12:181. 10.1186/1471-2288-12-181 23185978 PMC3552766

[14] Moher D , Shamseer L , Clarke M , et al . Preferred reporting items for systematic review and meta-analysis protocols (PRISMA-P) 2015 statement. Syst Rev 2015;4:1. 10.1186/2046-4053-4-1 25554246 PMC4320440

[15] Lizarondo L , Carrier J , Godfrey C . Chapter 8: Mixed methods systematic reviews. In: Aromataris E , Munn Z , eds. Joanna Briggs Institute reviewer’s manual [Internet]. The Joanna Briggs Institute, 2017. https://reviewersmanual.joannabriggs.org/

[16] Friedman AL , Kachur RE , Noar SM , et al . Health communication and social marketing campaigns for sexually transmitted disease prevention and control: what is the evidence of their effectiveness? Sex Transm Dis 2016;43:S83–101. 10.1097/OLQ.0000000000000286 26779691

[17] Veritas Health Innovation . Covidence systematic review software [Internet]. Melbourne, Australia: Veritas Health Innovation, 2021. www.covidence.org

[18] NIH . Study quality assessment tools for before-after (pre-post) studies with no control group [Internet], 2020. Available: https://www.nhlbi.nih.gov/health-topics/study-quality-assessment-tools

[19] Critical Appraisal Skills Programme . CASP: Qualitative Checklist [Internet], 2018. Available: https://casp-uk.net/wp-content/uploads/2018/01/CASP-Qualitative-Checklist-2018.pdf

[20] StataCorp . Stata statistical software: release 17. College station, TX: StataCorp LLC, 2020.

[21] Anderson EA , Eastman-Mueller HP , Henderson S , et al . Man up Monday: an integrated public health approach to increase sexually transmitted infection awareness and testing among male students at a Midwest university. J Am Coll Heal 2016;64:147–51. 10.1080/07448481.2015.1062768 26151349

[22] Buhrer-Skinner M , Muller R , Buettner PG , et al . Reducing barriers to testing for Chlamydia trachomatis by Mailed self-collected samples. Sex Health 2013;10:32–8. 10.1071/SH11065 23158104

[23] Chen MY , Karvelas M , Sundararajan V , et al . Evidence for the effectiveness of a Chlamydia awareness campaign: increased population rates of Chlamydia testing and detection. Int J STD AIDS 2007;18:239–43. 10.1258/095646207780658854 17509173

[24] Debattista J , Hayes M , Marshall P , et al . A trial of pharmacy-based testing for Chlamydia trachomatis using postal specimen kits. J Pharm Pract Res 2017;47:41–6. 10.1002/jppr.1221

[25] Friedman AL , Brookmeyer KA , Kachur RE , et al . An assessment of the GYT: get yourself tested campaign: an integrated approach to sexually transmitted disease prevention communication. Sex Transm Dis 2014;41:151–7. 10.1097/OLQ.0000000000000100 24521718 PMC6448774

[26] Friedman AL , Bozniak A , Ford J , et al . Reaching youth with sexually transmitted disease testing: building on successes, challenges, and lessons learned from local get yourself tested campaigns. Soc Mar Q 2014;20:116–38. 10.1177/1524500414530386 31749662 PMC6866650

[27] Gobin M , Verlander N , Maurici C , et al . Do sexual health campaigns work? an outcome evaluation of a media campaign to increase Chlamydia testing among young people aged 15-24 in England. BMC Public Health 2013;13:13:484. 10.1186/1471-2458-13-484 23683345 PMC3671151

[28] Gold J , Goller J , Hellard M , et al . Impact evaluation of a youth sexually transmissible infection awareness campaign using routinely collected data sources. Sex Health 2011;8:234–41. 10.1071/SH10082 21592439

[29] Guy R , Goller J , Leslie D , et al . No increase in HIV or sexually transmissible infection testing following a social marketing campaign among men who have sex with men. J Epidemiol Community Health 2009;63:391–6. 10.1136/jech.2008.077099 19179366

[30] Kwan KSH , Jachimowicz EA , Bastian L , et al . Online Chlamydia testing: an innovative approach that appeals to young people. Med J Aust 2012;197:287–90. 10.5694/mja11.11517 22938127

[31] Miller C , Hart G . Reduced disease prevalence at a sexually transmitted diseases clinic during a mass media campaign. Venereology 1995;8:37–42.

[32] Roston A , Suleta K , Stempinski K , et al . Get yourself tested 2011-2012: findings and prevalence of Chlamydia trachomatis and Neisseria gonorrhoeae at an urban public health system. Int J STD AIDS 2015;26:322–8. 10.1177/0956462414536541 24867819

[33] Rotblatt H , Montoya JA , Plant A , et al . There's no place like home: first-year use of the "I Know" home testing program for chlamydia and gonorrhea. Am J Public Health 2013;103:1376–80. 10.2105/AJPH.2012.301010 23327247 PMC4007891

[34] Wackett J . A theory-based initiative to reduce the rates of Chlamydia trachomatis infection among young adults in the Yukon. Can J Hum Sex 1998;7:347–56.

[35] Freeman E , Howell-Jones R , Oliver I , et al . Promoting chlamydia screening with posters and leaflets in general practice--a qualitative study. BMC Public Health 2009;9:383. 10.1186/1471-2458-9-383 19821964 PMC2766388

[36] Roush S , Aguinaldo J , Beauvoir C . Student perceptions and utilization of school-based health centers in Los Angeles: rsults of an STD prevention campaign. Sex Transm Dis 2016;43:S152.

[37] Nadarzynski T , Burton J , Henderson K , et al . Targeted advertisement of Chlamydia screening on social media: a mixed-methods analysis. Digit Health 2019;5:2055207619827193. 10.1177/2055207619827193 30746155 PMC6360644

[38] Garbers S , Friedman A , Martinez O , et al . Adapting the get yourself tested campaign to reach black and Latino sexual-minority youth. Health Promot Pract 2016;17:739–50. 10.1177/1524839916647329 27225216 PMC4980262

[39] Wilkins A , Mak DB . … sending out an SMS: an impact and outcome evaluation of the Western Australian Department of Health’s 2005 chlamydia campaign. Health Promot J Aust 2007;18:113–20. 10.1071/HE07113 17663646

[40] Patel A , Roston A , Suleta K . Get yourself tested 2011-2012: findings and rates of Chlamydia trachomatis and Neisseria gonorrhoeae at an urban public health system. Sex Transm Dis 2014;41:S52. 10.1177/0956462414536541 24867819

[41] Dowshen N , Lee S , Matty Lehman B , et al . IknowUshould2: feasibility of a Youth-Driven social media campaign to promote STI and HIV testing among adolescents in Philadelphia. AIDS Behav 2015;19:106–11. 10.1007/s10461-014-0991-9 25563502 PMC4495004

[42] van Liere GAFS , Dukers-Muijrers NHTM , van Bergen JEAM , et al . The added value of Chlamydia screening between 2008-2010 in reaching young people in addition to Chlamydia testing in regular care; an observational study. BMC Infect Dis 2014;14:612. 10.1186/s12879-014-0612-2 25403312 PMC4239384

[43] Spielberg F , Levy V , Kapur I , et al . O08.4 Online access to home STI specimen collection and E-prescriptions linked to public health - is a comparative effectiveness trial feasible? Sex Transm Infect 2013;89:A41.2–A41. 10.1136/sextrans-2013-051184.0128

[44] Kovaleski L , Aiern H , Beeston T . California’s take-home lesson: Is chlamydia (CT) and gonorrhea (GC) home testing a cost-effective way to provide saftey-net screening for young women? Sex Transm Dis 2018;45:S28–9.

[45] Lydié N , de Barbeyrac B , Bluzat L , et al . Chlamyweb study I: rationale, design and acceptability of an Internet-based Chlamydia testing intervention. Sex Transm Infect 2017;93:179–87. 10.1136/sextrans-2015-052511 28258251

[46] McDaid LM , Lorimer K . P5.044 A proactive approach to onnline chlamydia screening: Qualitative exploration of young men’s perspectives of the Barriers and facilitators. Sex Transm Infect 2013;89:A348.1–348. 10.1136/sextrans-2013-051184.1088

[47] Lee JGL , Landrine H , Martin RJ , et al . Reasons for caution when emphasizing health disparities for sexual and gender minority adults in public health campaigns. Am J Public Health 2017;107:1223–5. 10.2105/AJPH.2017.303883 28700295 PMC5508158

[48] Cho H , Salmon CT . Unintended effects of health communication campaigns. J Commun 2007;57:293–317. 10.1111/j.1460-2466.2007.00344.x

[49] Wakefield MA , Loken B , Hornik RC . Use of mass media campaigns to change health behaviour. Lancet 2010;376:1261–71. 10.1016/S0140-6736(10)60809-4 20933263 PMC4248563

[50] Clar C , Dyakova M , Curtis K , et al . Just telling and selling: current limitations in the use of digital media in public health: a scoping review. Public Health 2014;128:1066–75. 10.1016/j.puhe.2014.09.009 25443388

[51] Park A , Eckert TL , Zaso MJ , et al . Associations between health literacy and health behaviors among urban high school students. J Sch Health 2017;87:885–93. 10.1111/josh.12567 29096408 PMC5669371

[52] Low N , Bender N , Nartey L , et al . Effectiveness of Chlamydia screening: systematic review. Int J Epidemiol 2009;38:435–48. 10.1093/ije/dyn222 19060033

