# Supplementary material for: What is the effectiveness of community-based health promotion campaigns on chlamydia screening uptake in young people and what barriers and facilitators have been identified? A mixed-methods systematic review
Source: Sex Transm Infect. 2021 Aug 26;98(1):62–9. doi: 10.1136/sextrans-2021-055142 (PMC8785066; doi:10.1136/sextrans-2021-055142)
Supplement: Supplementary data [file sextrans-2021-055142supp001.pdf]

Appendix 1 Example search strategy - MEDLINE

1 Chlamydia Infections/ or Chlamydia/ or Chlamydia trachomatis/

2 chlamydia.ti,ab.

3 1 or 2

4 (campaign* or encourag*).ti,ab.

5 advert*.ti,ab.

6 social media.ti,ab.

7 advertising/

8 social media/

9 exp Internet/

10 social networking/

11 internet.ti,ab.

12 (social network* or Twitter or Facebook or Foursquare or Qzone or Tumblr or Instagram or Google or WhatsApp or Messenger or Meetup or Wechat or Linkedin or Snapchat or "snap chat" or Pinterest or Sina Weibo or Tik Tok or Tiktok or YouTube or Reddit or Baidu Tiebaor or Viber or VKontakte or XING or Tinder or web or www).ti,ab.

13 Internet-based intervention/

14 text messaging/

15 (SMS or mobile or smartphone* or app*).ti,ab.

16 cellphone*.ti,ab.

17 cell* phone*.ti,ab.

18 exp cell phone/

19 (tv or television or digital or video* or dvd* or radio or broadcast* or media).ti,ab.

20 telemedicine/

21 mHealth.ti,ab.

22 (flyer* or Poster* or newspaper* or workshop* or discussion* or leaflet*).ti,ab.

23 Posters as Topic/

24 health education/ or consumer health information/ or health fairs/ or patient education as topic/ or sex education/

25 marketing of health services/

26 health education.ti,ab.

27 exp health Promotion/

28 health behavior/

29 social marketing/

30 behavioral medicine/

31 exp patient Acceptance of healthcare/

32 or/4-31

33 3 and 32

34 limit 33 to humans
