# Supplementary material for: What is the effectiveness of community-based health promotion campaigns on chlamydia screening uptake in young people and what barriers and facilitators have been identified? A mixed-methods systematic review
Source: Sex Transm Infect. 2021 Aug 26;98(1):62–9. doi: 10.1136/sextrans-2021-055142 (PMC8785066; doi:10.1136/sextrans-2021-055142)

Supplementary materials:

Supplemental information on quality assessment of quantitative studies:

Two items were considered not applicable for any of the included studies (blinding of assessors and accounting for whole group-level data), leaving ten assessed items.[18] All items were judged to be of similar importance to study quality therefore if a study had the same determination of risk of bias (low, medium, high) in five or more items then this determination was ascribed to the whole study. If two adjacent bias domains scored equally (eg. four low, four medium and two high), the overall quality assessment was taken to be the higher risk of the two, and if 'low' and 'high' scored equally (eg. three low, four medium, three high) then the study was assessed as at medium risk.

Supplementary figure 1: Relative change in test count (males). This image is not reproduced from another source and has been created by the review team for the current publication.

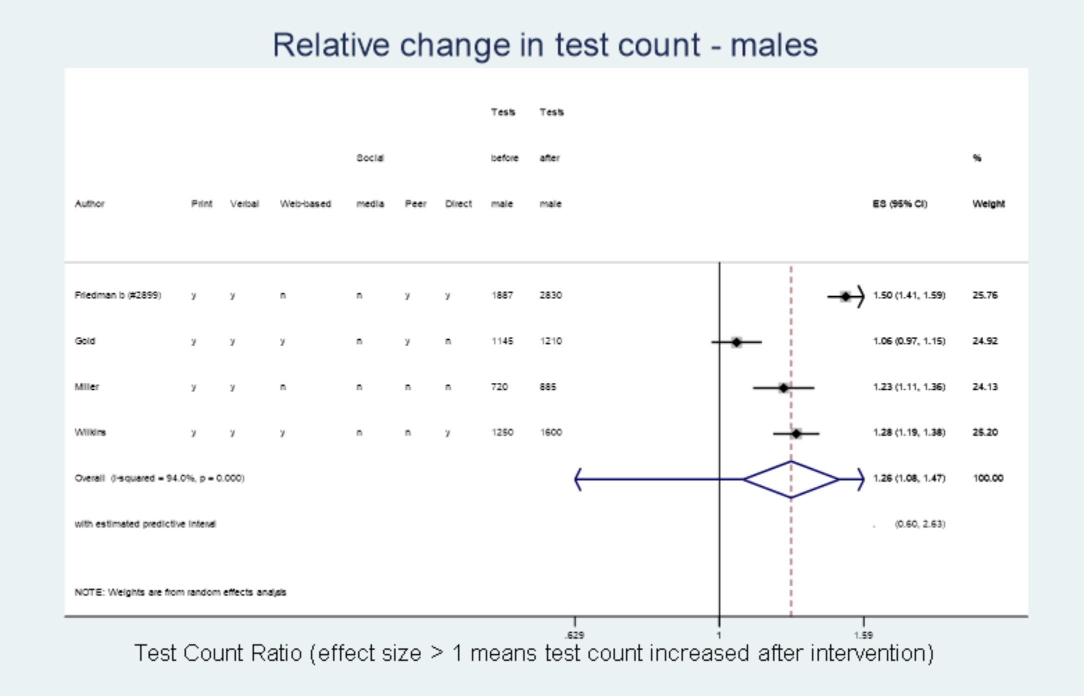

Supplementary figure 2: Relative change in test count (females). This image is not reproduced from another source and has been created by the review team for the current publication.

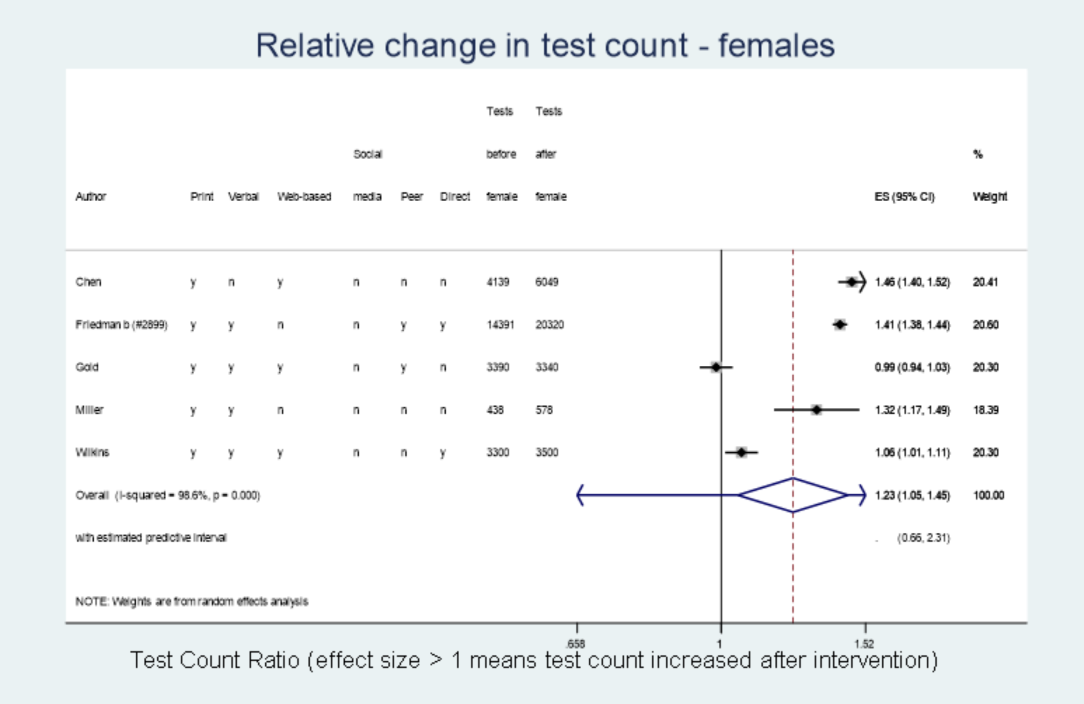

Supplementary figure 3: Relative change in positive test count (all). This image is not reproduced from another source and has been created by the review team for the current publication.

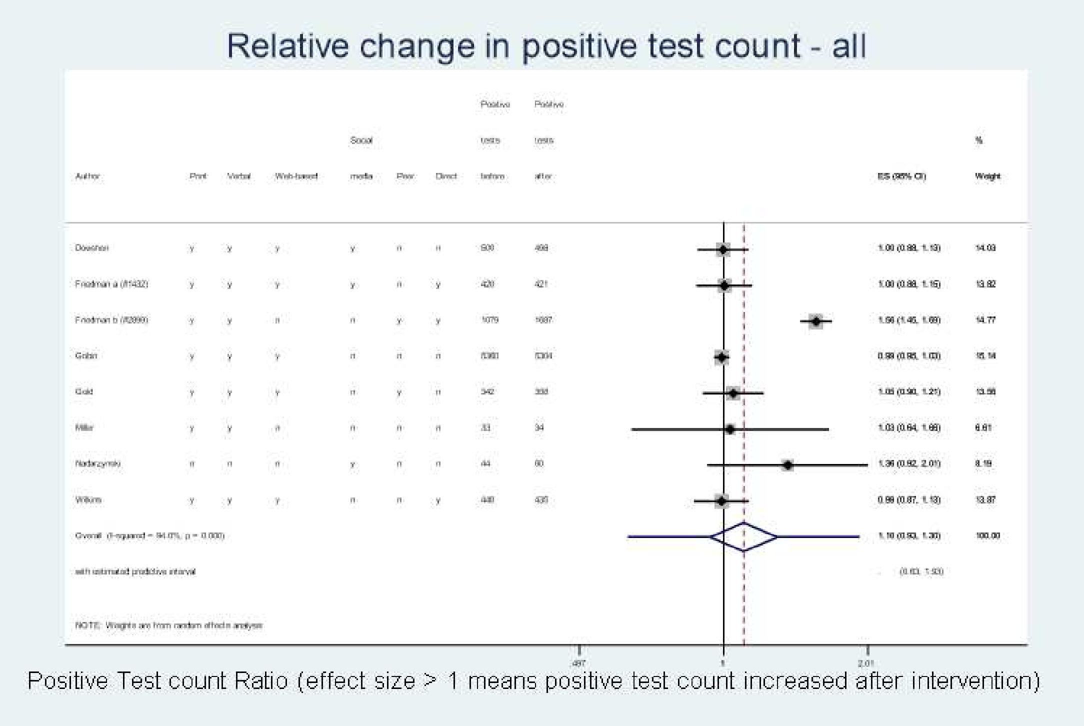

Supplementary figure 4: Relative change in positive test count (males). This image is not reproduced from another source and has been created by the review team for the current publication.

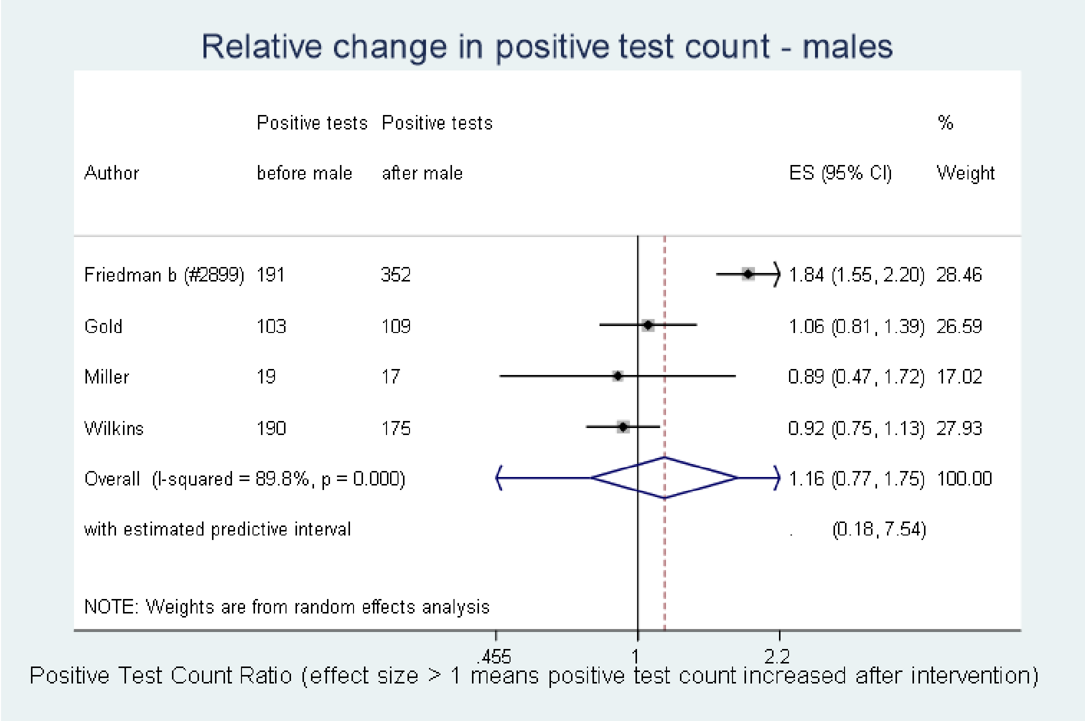

Supplementary figure 5: Relative change in positive test count (females). This image is not reproduced from another source and has been created by the review team for the current publication.

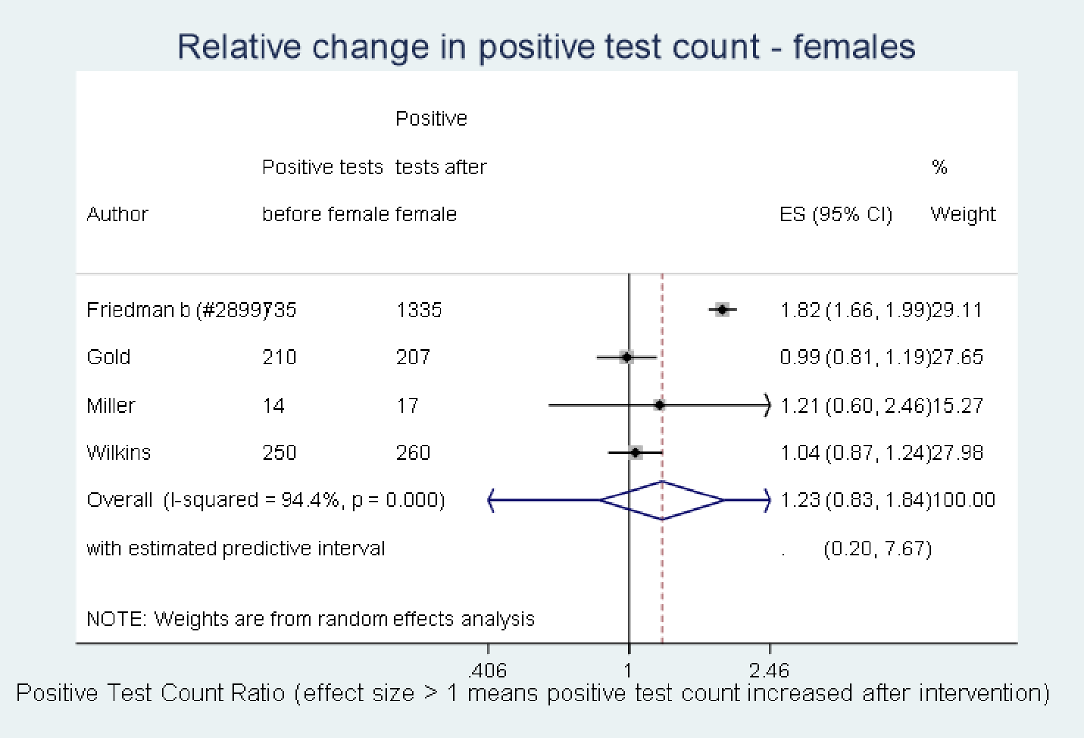

Supplementary figure 6: Relative change in positivity rate (males). This image is not reproduced from another source and has been created by the review team for the current publication.

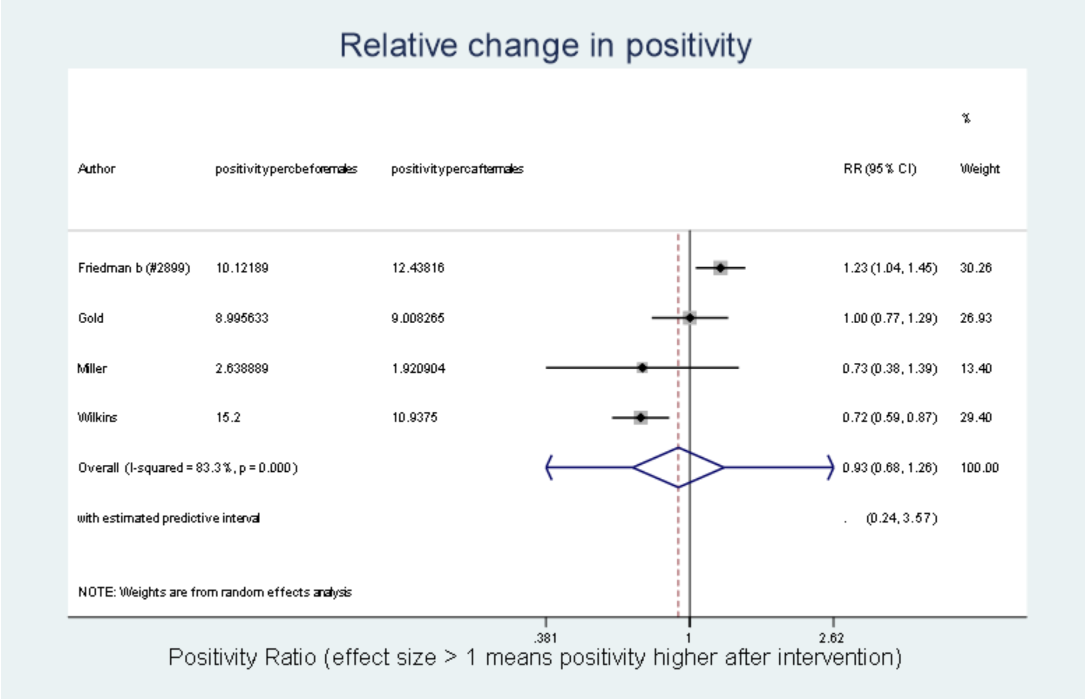

Supplementary figure 7: Relative change in positivity rate (females). This image is not reproduced from another source and has been created by the review team for the current publication.

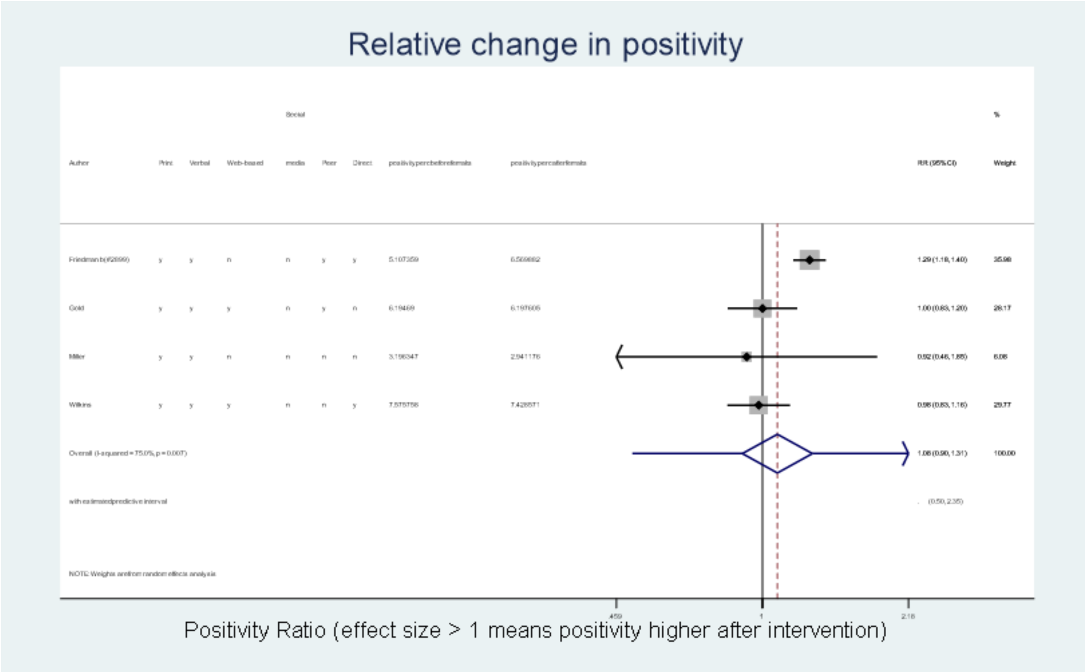

Supplement: Supplementary data [file sextrans-2021-055142supp004.pdf]
