# Supplementary material for: What is the effectiveness of community-based health promotion campaigns on chlamydia screening uptake in young people and what barriers and facilitators have been identified? A mixed-methods systematic review
Source: Sex Transm Infect. 2021 Aug 26;98(1):62–9. doi: 10.1136/sextrans-2021-055142 (PMC8785066; doi:10.1136/sextrans-2021-055142)
Supplement: Supplementary data [file sextrans-2021-055142supp003.pdf]

Appendix : Quotations to support qualitative findings

Quotations have been removed and added to this appendix owing to word limit constraints.

Targeting:

> "*There was a campaign that was put out, by the New York Health Department that was controversial about the scare tactics that they used, that was kind of offending the LGBTQ community. Basically saying, just because you're a man of color, you're automatically going to be positive anyway*"(Young Person) [38]

Quality of message and materials:

> "*The leaflets for the patient are absolutely superb, very self-explanatory; they are quite small and very necessary because they're all waiting to go in to see the doctor. Its nice print, it's nicely put and nice to read it's so easy to explain*" (Staff Member)[35]

> "*I think like bright and colourful, certainly draws your attention much quicker and I think, as well, it gives the impression more effort has been put into it*" (Young Person)[37]

Language:

> "*Because people come with different languages, if you say some [thing] even if it's in simple English they would not understand the meaning of it. Preferably I would like to have them in as many languages as possible, but it's not really viable from the PCT point [of view]. They cannot have an enormous amount of languages just for chlamydia screening, there's other things as well*" (Staff Member)[35]

Anonymity:

> "*If it could be seen that I was viewing it like that would deter me because I wouldn't want people to know*" (Young Person)[37]

Use of technology:

> "*Our generation are on their phones a lot using social media, so online promotion would be useful*" (Young Person)[37]

> "*I don't know if this national advertising works, [use] the text via mobile, that would be a good point, they've all got mobile phones*" (Staff Member)[35]

Relevance to the young person:

> "*I think [messaging] needs to be more generic - that is, reaching out to different crowds. We're obviously talking about sex, the words are talking about sex; the picture doesn't have to be about sex. You could have a video game controller, and someone who likes video games is always going to look at that advertisement, but they're also going to get the message*" (Young Person)[38]

> "*I wouldn't want some 58-year-old woman telling me about sex because I'd feel like I can't relate to her, or that she can't relate to my situation. I'd prefer someone probably a female, within around a 5-year age gap in the advert*" (Young Person)[37]

Variety of options for testing:

"*I think maybe advertising on the website and places where young people were going to look and read, as well as having the whole list of where they can get access to everything, not just one service*" (Staff member)[35]
