# Supplementary material for: What is the effectiveness of community-based health promotion campaigns on chlamydia screening uptake in young people and what barriers and facilitators have been identified? A mixed-methods systematic review
Source: Sex Transm Infect. 2021 Aug 26;98(1):62–9. doi: 10.1136/sextrans-2021-055142 (PMC8785066; doi:10.1136/sextrans-2021-055142)
Supplement: Supplementary data [file sextrans-2021-055142supp002.pdf]

Appendix 2 Campaign characteristics identified from qualitative analysis and outcomes

Author (year)		Characteristics thought to be facilitators of testing						Testing outcomes: Relative change (95% CI)		
	Targeting	Quality	Language	Anonymity	Technology	Relevance	Test options	Test count	Positive test count	Positivity rate
Anderson (2016)	X							-	-	-
Buhrer-Skinner (2013)	X			X			X	-	-	-
Chen (2007)	X							-	-	-
Debattista (2017)	X							-	-	-
Dowshen (2015)	X			X	X	X	X	1.01 (0.97, 1.06)	1.00 (0.88, 1.13)	0.98 (0.88, 1.11)
Friedman[a] (2014)	X			X	X	X	X	1.15 (1.11, 1.19)	1.00 (0.88, 1.16)	0.87 (0.77, 1.00)
Friedman[b] (2014)	X			X	X	X	X	1.41 (1.38, 1.44)	1.56 (1.45, 1.69)	1.11 (1.03, 1.19)
Garbers (2016)	X					X	X	1.16 (0.95, 1,41)	-	-
Gobin (2013)	X					X		1.27 (1,26, 1.29)	0.99 (0.95, 1.03)	-
Gold (2011)	X					X		1.04 (1.00, 1.09)	1.05 (0.90, 1.21)	0.78 (0.75, 0.81)
Kwan (2012)	X			X				3.33 (1.58, 7.02)	-	-
Kwan 2 (2012)	X			X				1.78 (1.07, 2.97)	-	-
Miller (1995)	X							1.29 (1.13, 1.48)	1.03 (0.64, 1.66)	0.82 (0.51, 1.31)
Nadarzynski (2019)	X			X	X	X		-	1.36 (0.82, 2.01)	1.05 (0.73, 1.52)
Roston (2015)	X		X					1.44 (1.33, 1.56)	-	-
Rotblatt (2013)	X		X	X		X		-	-	-
Wackett (1998)	X		X			X		1.15 (1.04, 1.26)	-	-
Wilkins (2007)	X				X			1.12 (1.08,1.17)	0.99 (0.87,1.13)	0.88 (0.81,1.07)
